# CD46 TREM1 regulates the autophagy marker LC3B ATG5 in oral squamous cell carcinoma

**DOI:** 10.3389/fonc.2025.1579282

**Published:** 2025-05-22

**Authors:** Xiaodan Liu, Fumin Zheng, Meihua Gao, Yuanfei Wang, Xiaofang Lv, Beibei Cong, Wanchun Wang

**Affiliations:** ^1^ School of Stomatology, Binzhou Medical University, Yantai, China; ^2^ Department of Oral Mucosal Diseases, Qingdao Stomatological Hospital Affiliated to Qingdao University, Qingdao, China; ^3^ School of Stomatology, Qingdao University, Qingdao, China

**Keywords:** squamous cell carcinoma, CD46/TREM1, regulation mechanism, LC3B, ATG5

## Abstract

**Objective:**

This study aims to explore the regulatory effect of abnormal expression of CD46 and TREM1 on LC3B and ATG5 and to provide new targets for the molecular mechanism and treatment of OSCC.

**Methods:**

Human oral squamous cell carcinoma cells CAL-27 were selected for *in vitro* culture. For related research, the CD46 shRNA interference test, immunohistochemistry (IHC), flow cytometry, qRT-PCR detection, Western blot, and tumor-bearing animal models were used.

**Results:**

*In vitro* cell experiments showed that CD46 and TREM1 were highly expressed on the surface of OSCC cells, while the expression of LC3B and ATG5 was significantly decreased. Compared with the control group (SC-shRNA), the CD46 shRNA group could effectively reduce the expression of CD46 in OSCC cells and increase the expression of autophagy and apoptosis protein (*P*<0.01). *In vivo* experiments showed that the tumor volume of the shRNA group was significantly smaller than that of the SC-shRNA group (*P*<0.01), the expression of CD46 and TREM1 was decreased considerably, and the expression of LC3B and ATG5 was higher (*P*<0.01).

**Conclusion:**

OSCC cells have high expression of CD46 and TREM1, while low expression of LC3B and ATG5, and the autophagy apoptosis signal is weakened. Interfering with CD46 can up-regulate the expression of autophagy apoptosis genes, reduce the tumor inflammatory microenvironment, induce the apoptosis of OSCC cells, and inhibit the proliferation and metastasis of OSCC. This study provides a new idea for the mechanism and targeted therapy of OSCC and has important theoretical significance and clinical application value.

## 1 Introduction

Oral squamous cell carcinoma (OSCC) is a standard head and neck malignant tumor that seriously endangers human health, the predilection sites include the tongue, gums, cheeks, etc. (1). Studies have shown that the number of patients in most countries has declined in recent years, but the mortality rate of the disease is still on the rise. The treatment methods are mainly surgery, combined with chemotherapy and radiotherapy, but the prognosis is poor and easy to relapse (2, 3). At present, many scholars are committed to finding targeted drugs for specific targets of OSCC to inhibit or slow down the occurrence and development of OSCC. Therefore, exploring the molecular mechanism of OSCC, finding new targets, and developing specifically targeted drugs is imperative.

Many studies have shown that the mechanism of tumorigenesis is mainly related to the tumor microenvironment affected by autophagy and inflammation (2, 4). Autophagy is a process in which cells undergo self-degradation and clearance, and it is a method for cells to maintain homeostasis (5, 6). It is necessary for cell survival, including a variety of autophagy-related genes and regulated by them (7), including LC3B (5), ATG5, etc. Inflammation is the mechanism of the body’s defense against the invasion of foreign microorganisms and various pathogens. The inflammatory pathways of tumors mainly include endogenous and exogenous pathways, which are primarily mediated by tumor-associated macrophages and T lymphocytes, resulting in the corresponding inflammatory factors such as TNF-α, IL-6, and so on (1), and then form the inflammatory microenvironment of tumors, which provides the basis for the occurrence and development of tumors.

By combining bioinformatics screening with immunohistochemical validation, we identified hub genes that could advance biomarker discovery and targeted therapy development in OSCC. Based on data from The Cancer Genome Atlas (TCGA),we explore the regulatory effect of abnormal expression of CD46 and TREM1 on LC3B and ATG5 and to provide new targets for the molecular mechanism and treatment of OSCC. Our study found that CD46 and TREM1 were highly expressed on the surface of OSCC tissues, and the apoptotic protein Bax was decreased. It is speculated that CD46 and TREM1 may inhibit autophagic apoptotic genes LC3B and ATG5 by mediating the activation of the inflammatory cancer chain, thus weakening the apoptotic signal and leading to the proliferation and escape of cancer cells. The purpose of this study is to explore the regulatory effect of CD46 and TREM1 on LC3B and ATG5 and the inhibitory effect on the progression of inflammatory cancer from the cellular, molecular, and genetic levels through *in vitro* and *in vivo* experiments and to open a new way for the molecular mechanism and targeted diagnosis and treatment of clinical OSCC.

## 2 Materials and methods

### 2.1 Data sources and processing

RNA sequencing data and clinical annotations for head and neck squamous cell carcinoma (HNSC) were retrieved from the TCGA-HNSC cohort (https://www.cancer.gov/tcga) via the UCSC Xena platform (http://xena.ucsc.edu/) on 1 January 2025. The dataset included 518 tumor specimens and 44 normal tissue samples from 530 patients (**Supplementary Table S1**). The R package limma (version 3.40.6) was utilized to identify differentially expressed genes (DEGs) between tumor and normal tissues. Genes were considered DEGs if they met the criteria of |log2(fold change)| > 1.3 and P < 0.05. Volcano plots illustrating the DEGs were generated using the ggplot2 package in R. DEG identifiers were converted to gene symbols using org.Hs.eg.db (v3.10) in R, followed by GO-based functional enrichment analysis via clusterProfiler (v3.14.3). GO terms with P < 0.05 were deemed significant. The expression profiles of hub genes in HNSC were analyzed via the GEPIA web server (http://gepia.cancer-pku.cn/). The prognostic value of these genes was evaluated through Kaplan-Meier survival analysis using the survival package (v3.2-7) in R.

### 2.2 Sample selection

A total of 29 cases of paraffin-embedded tissue specimens of oral squamous cell carcinoma confirmed by pathology from 2020.05 to 2023.09 in the Affiliated Hospital of Qingdao University were selected as the experimental group, including 15 males and 14 females aged 40–89 years (Ethical number: QDU-HEC-2022127) (Patient specific clinical information is displayed in **Supplementary Table S2**). Another 10 cases of clinically extracted wisdom teeth without inflammatory reaction were taken, and the periodontal tissue was cut and embedded in paraffin as the normal control group (2023.09 to 2023.10). At the same time, 10 cases of clinically extracted wisdom teeth with inflammatory reaction were collected, and the periodontal tissue was cut and embedded in paraffin as the inflammatory control group (2023.09 to 2023.10).

### 2.3 CD46 shRNA interference experiment

CD46 interference experiment: 2.5 mL CAL-27 cells (5 × 10^4^ cells/mL) were added to 6-well plates and cultured in 5% CO_2_ incubator for 24 h (cell density reached 20 - 30%). According to the MOI value (MOI = 10), SC-shRNA, shRNA virus, and infection solution (Jikai gene) were added at 1 × 10^7^ TU/mL concentration. After 16 h, the medium was replaced with complete medium. After 72 h, 5 μg/mL puromycin was added for screening for 48 h to obtain the corresponding stable strain (infection rate > 80%) for subsequent experiments.

### 2.4 Immunohistochemistry

The expression of CD46, TREM1 and LC3B ATG5 on the surface of OSCC cells was observed by immunohistochemical staining (IHC) (8). The experimental steps were carried out according to the steps of the Zhongshan Jin Qiao detection kit.

### 2.5 Complement binding experiment

The inhibitory effect of CD46 on complement was observed.CAL-27 (5 × 10^4^ cells/mL) 100 μL was added to a 96-well plate, and 5 duplicate wells were set for each group. The cells were cultured in a 5% CO_2_ incubator at 37°C for 24 h, SC-shRNA and shRNA virus vectors with a concentration of 1 × 10^7^ TU/mL were added. After transfection for 72 h, 20% serum (HS) was added to each well for co-culture for 2 h, and 10% CCK-8 detection solution was added to each well. The absorbance at 450 nm (OD value) was detected by the microplate reader.

### 2.6 LDH assay

Detection of apoptosis. According to the manufacturer’s instructions, (Jiang Lai Biological), commercial kits were used to detect the lactate dehydrogenase (LDH) level in the cell supernatant of CAL-27 treated with SC-shRNA and shRNA, respectively.

### 2.7 Flow cytometry: observation of apoptosis

According to the Annexin V-APC/PI apoptosis detection kit operation steps. The above stable transfected cells were routinely digested, and 5 × 10^5^ cells were collected. The supernatant was discarded by centrifugation, washed with PBS 3 times, and 100 μL diluted 1 × Annexin V Binding Buffer was added to re-suspend the cells. After mixing, the cells were incubated in the dark for 15–20 min, and 400ul diluted 1× Annexin V Binding Buffer was added and mixed well.

### 2.8 qRT-PCR detection

Quantitative reverse transcription-polymerase chain reaction (qRT-PCR) was used to observe the changes of CD46, LC3B, ATG5, Bax, Bcl-2, Caspase-3 and tumor necrosis factor TNF-α after CAL-27 was treated with SC-shRNA and shRNA. Total RNA was extracted from cells using TRIzol reagent. The RNA was treated according to the instructions of PrimeScript™RT reagent Kit and TB Green^®^ Premix Ex Taqr II (Tli RNaseH Plus) kit, and the relative expression level was compared with glyceraldehyde-3-phosphate dehydrogenase (GAPDH) as an internal reference. △Ct was recorded automatically, and the relative expression of each gene was calculated by 2^−△△Ct^ method. All primer sequences used in this study were provided by Shanghai Sangon (**Table 1**).

**Table 1:** PCR primer sequences for autophagy-related (LC3B, ATG5) and apoptosis-related (Bcl-2, Bax, Caspase-3) genes, along with inflammatory marker (TNF-α) and reference gene (GAPDH).

Group	Sequence Information
CD46	F: ACCAACATTTGAAGCTATGGAGC; R: GCCATGTATGATTCCGATCACAA;
LC3B	F: AGCGTCTCCACACCAATCTCAG; R: ACAATTTCATCCCGAACGTCTCC;
ATG5	F: GAATATGAAGGCACACCACTGAAATG; R: GTACTGTGATGTTCCAAGGAAGAGC;
Bcl-2	F: CGAGTGGGATGCGGGAGATG; R: CGGGATGCGGCTGGATGG;
Bax	F: CCAAGAAGCTGAGCGAGTGTC; R: GTCCACGGCGGCAATCATC;
Caspase-3	F: GAACTGGACTGTGGCATTGAGAC; R: AATAATAACCAGGTGCTGTGGAGTATG;
TNF-a	F: CTCATCTACTCCCAGGTCCTCTTC; R: CGATGCGGCTGATGGTGTG;
GAPDH	F: TTGGTATCGTGGAAGGACTCTA; R: TGTCATATTTGGCAGGTT;

### 2.9 Western blot

The expression of CD46, LC3B, p62, ATG5, Bax, Bcl-2, Caspase-3 and TNF-αin CAL-27 treated with SC-shRNA and shRNA was observed by Western blot. The protein was extracted according to the RIPA lysis buffer instructions, and the protein concentration was determined using the BCA kit. The 4 × loading buffer was added and boiled in boiling water for 5 min, and the sample was retained for use (7). The electrophoresis gel was prepared by using 12.5% one-step PAGE gel rapid preparation kit. The sample volume of each well protein was 60 μg, marker 5 μL; tris/glycine/SDS electrophoresis buffer instant granules 150 V, 45min electrophoresis; free ice bath rapid transfer membrane buffer instant granules 400 mA, 45 min under the condition of electric transfer; 5% skimmed milk powder closed 2 h; TBST was washed three times, and the primary antibody was diluted with antibody diluent, and incubated overnight at 4°C, including rabbit anti-CD46, LC3B, p62, ATG5, Bax, Bcl-2, Caspase-3, TNF-α, β-actin monoclonal antibodies; TBST was washed three times, and goat anti-rabbit secondary antibody was incubated at room temperature for 2 h. TBST was washed three times, the ECL hypersensitivity developer was configured according to the instructions, and the analysis was performed using a tableting imager (G. BLOT). Finally, Image J software was used to analyze the bands quantitatively.

### 2.10 Transmission electron microscopy

CAL-27 cells subjected to treatment were initially fixed with 4% glutaraldehyde, followed by secondary fixation with 1% osmium tetroxide. The samples were then dehydrated through a graded ethanol series (70%, 90%, and absolute ethanol). Then, the samples were transferred into a mixture of Epon 812 epoxy resin and acetone for infiltration, followed by embedding in pure resin for polymerization. After embedding, ultra-thin sections (70–80 nm) were prepared and subsequently analyzed using a TEM (JEOL, Japan) operated at an accelerating voltage of 80 kV.

### 2.11 *In vivo* experiment

Construction of tumor-bearing mouse tumor model (9), to verify the effect of CD46 on OSCC apoptosis. Twenty 6-week-old nulliparous male nude mice (weighing about 16–20 g) were randomly divided into four groups and acclimatised and fed for one week. The animal experiments were conducted following the approved protocol by the Animal Ethics Committee of Qingdao University (Ethical number: QDU-AEC-2022232). The tumor was successfully implanted on the 7th day, and the tumor size was observed regularly. The calculation formula was volume (mm^3^) = d^2^ × D/2, where d and D were the shortest and longest diameter (10). On the 7th and 14th days, the tumor size was recorded by taking photos (11). At the end of the experiment, the mice were sacrificed by intraperitoneal injection of excessive pentobarbital (75 mg/kg) (11). The tumor was weighed. 1% formaldehyde fixed, paraffin-embedded sections, HE staining, microscopic observation. The expression of CD46, TREM1, LC3B and ATG5 in each group was detected by IHC (10, 12).

### 2.12 Statistical analysis

The results were expressed as the mean ± standard error of each group. All data were statistically analyzed using GraphPad Prism 9.5 software. Comparison between the two groups using t-test, *P* < 0.05 was considered statistically significant.

## 3 Result

### 3.1 Identification of CD46 and TREM1 as prognostic hub genes in HNSC through integrated transcriptomic and survival analysis

**Figure 1:**
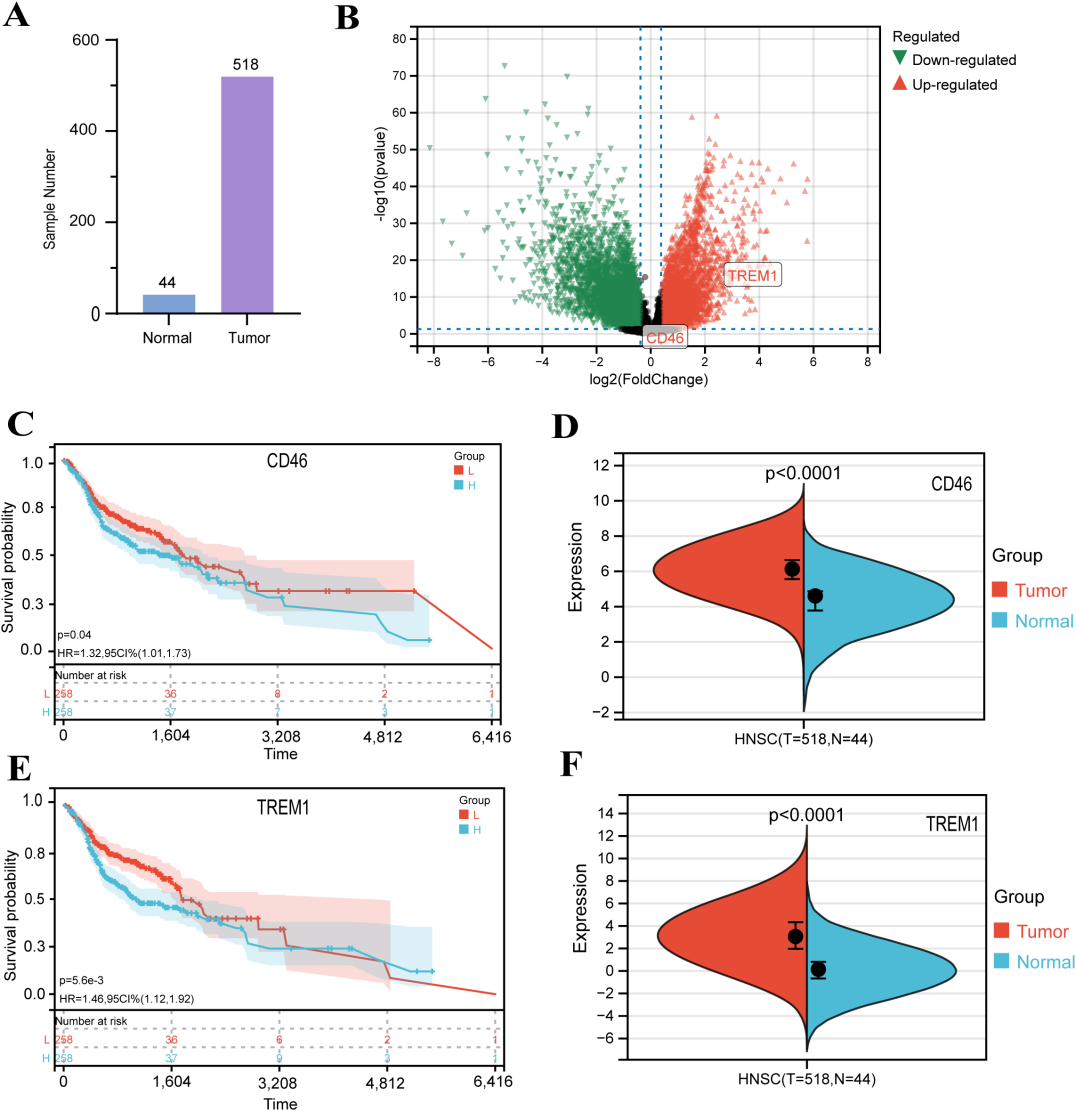
Identification and validation of DEGs in HNSC: **(A)** Principal component analysis distinguishing 44 normal and 518 HNSC samples. **(B)** Volcano plot of DEGs. Red: upregulated genes in HNSC (|log2FC| > 1.3, P < 0.05); green: downregulated genes; gray: non-significant genes. **(C, E)** Kaplan-Meier survival curves for HNSC patients stratified by CD46 and TREM1 expression levels (log-rank test, P < 0.01). **(D, F)** Comparison of CD46 and TREM1 expression between normal and tumor tissues in the GEPIA database.

Our study utilized transcriptomic data from 44 normal and 518 HNSC tumor samples (**Figure 1A**, **Supplementary Table S1**). Differential expression analysis identified 9,276 DEGs (4,404 upregulated and 4,872 downregulated) in tumors compared to normal tissues (**Figure 1B**). To elucidate their functional roles, GO enrichment analysis was performed. Upregulated DEGs were enriched in processes including cellular developmental regulation, differentiation, and cell cycle progression (**Supplementary Figure S1**), whereas downregulated DEGs clustered in pathways related to ion transport, metabolic regulation, and oxidoreductase activity (**Supplementary Figure S2**). These findings align with established molecular hallmarks of HNSC, supporting the reliability of our dataset. Leveraging TCGA clinical and expression data, we further evaluated associations between DEG expression levels and patient survival outcomes to prioritize prognostic biomarker candidates. The survival analysis revealed that elevated expression of CD46 and TREM1 is significantly associated with poor prognosis in HNSC. Specifically, high CD46 expression correlated with reduced overall survival (OS) compared to low expression (log-rank p = 0.04; HR = 1.32, 95% CI: 1.01–1.73), suggesting a potential role in disease progression. Notably, TREM1 demonstrated a stronger prognostic impact, with high expression linked to markedly poorer OS (p = 0.0056; HR = 1.46, 95% CI: 1.12–1.92). These findings highlight both genes as candidate biomarkers for risk stratification, with TREM1 showing greater statistical significance and clinical relevance (**Figure 1C, E**). In addition, it can be seen that the expression of CD46 and TREM1 in tumors is higher than that of normal tissues (**Figure 1D, F**). Thus, we defined these genes as ‘final’ hub genes.

### 3.2 The expression of CD46, TREM1, LC3B and ATG5 in OSCC tissues and cell surface

**Figure 2:**
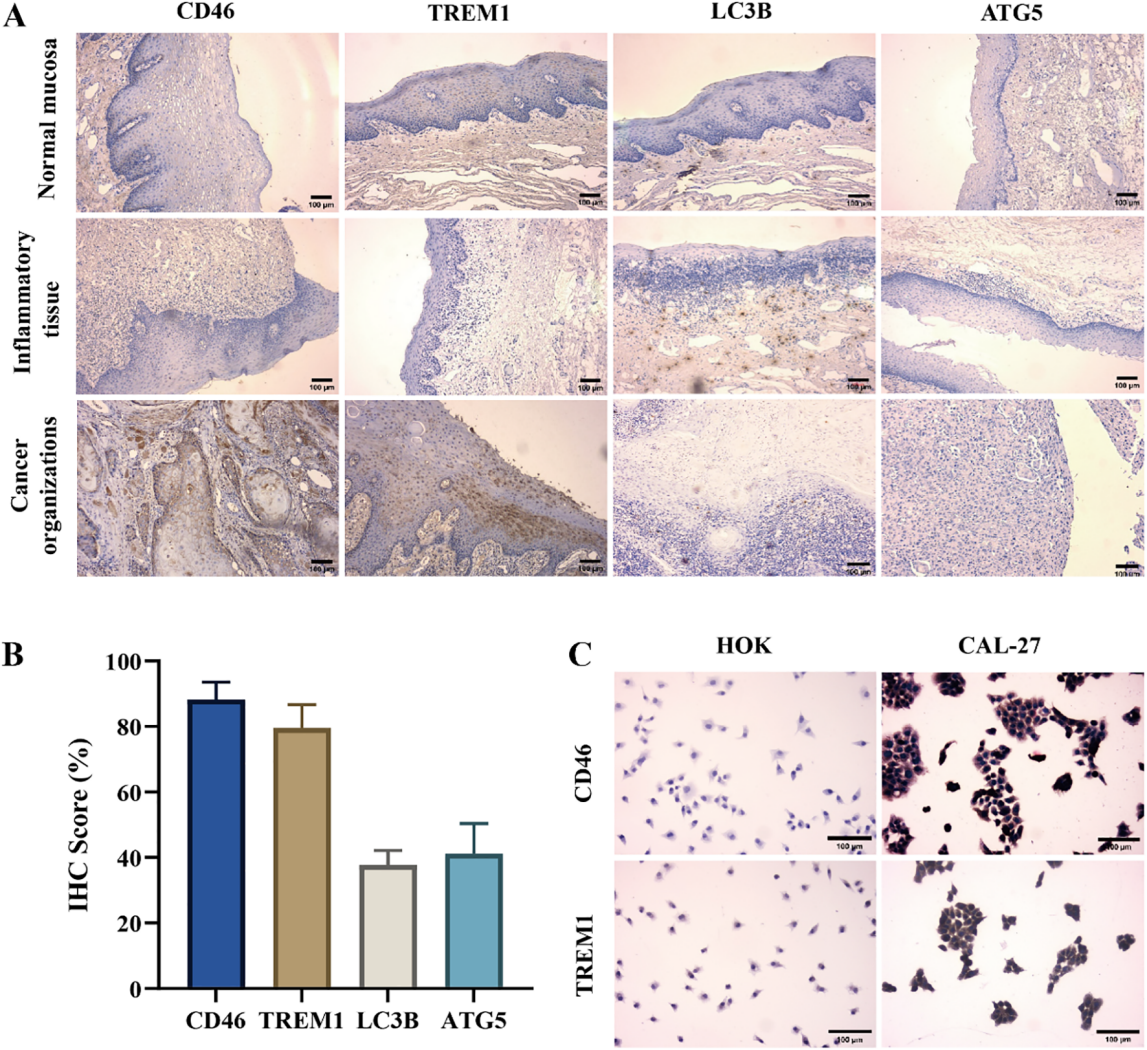
High expression of CD46 and TREM1 in OSCC tissues and cell surface: **(A)** The expression of CD46, TREM1, LC3B and ATG5 on the surface of three tissues (10 ×). **(B)** Statistical graphs of positive rates of CD46, TREM1, LC3B and ATG5 in tumor immunohistochemistry in 29 OSCC patients. **(C)** The expression of CD46 and TREM1 on HOK and CAL-27 (20 ×).

The immunohistochemistry results showed that the expression of CD46 and TREM1 in cancer tissues was significantly higher than that in normal mucosa and inflammatory tissues (*P* < 0.01). The expression of autophagy-related genes LC3B and ATG5 decreased (**Figure 2AB**) (*P* < 0.01). Consistent with the results of cell experiments (**Figure 2C**). The above results indicate that CD46 and TREM1 can promote the proliferation and escape of OSCC by inhibiting autophagy and apoptosis genes.

### 3.3 The expression of LC3B and ATG5 in OSCC cells after CD46 interference

**Figure 3:**
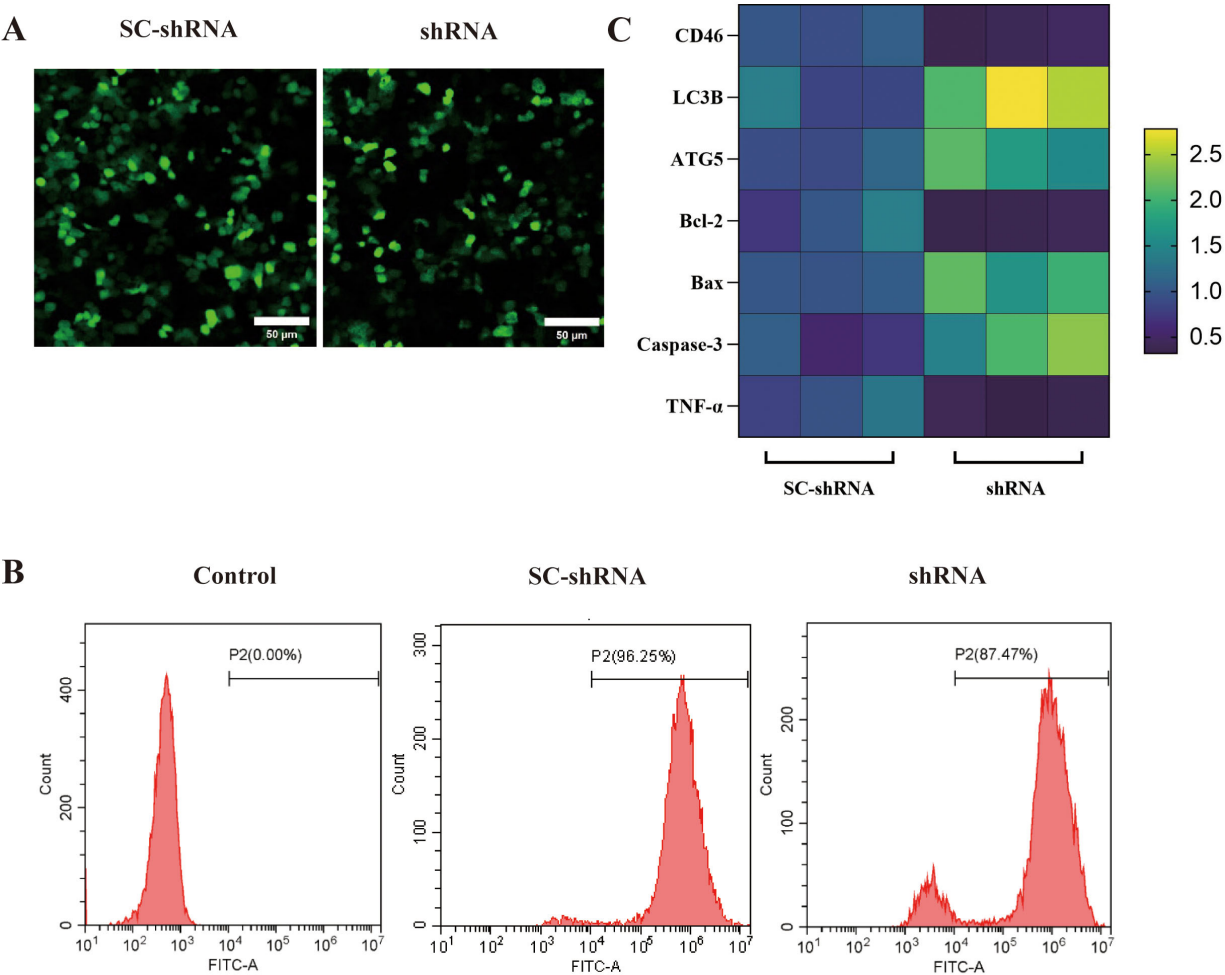
Effect of knockdown of CD46 on autophagy and apoptosis of OSCC: **(A)** SC-shRNA and shRNA were successfully transfected into CAL-27 (20 ×). **(B)** Flow cytometry was used to detect the transfection rate of different groups of cells (> 80%). **(C)** PCR was used to detect the heat map of the incremental changes of each gene after CD46 knockdown.

The expression of CD46 mRNA in OSCC cells was detected by flow cytometry and qRT-PCR. The results showed that the level of CD46 in the shRNA group was lower than that in the SC-shRNA group, and the expression of autophagy-related apoptosis-related genes was increased, indicating that CD46 was successfully knocked down. Knockdown of CD46 activated OSCC autophagy and promoted cancer cell apoptosis. The difference between the groups was very significant (**Figure 3**) (*P* < 0.01).

### 3.4 The effect of CD46 interference on OSCC cell apoptosis

**Figure 4:**
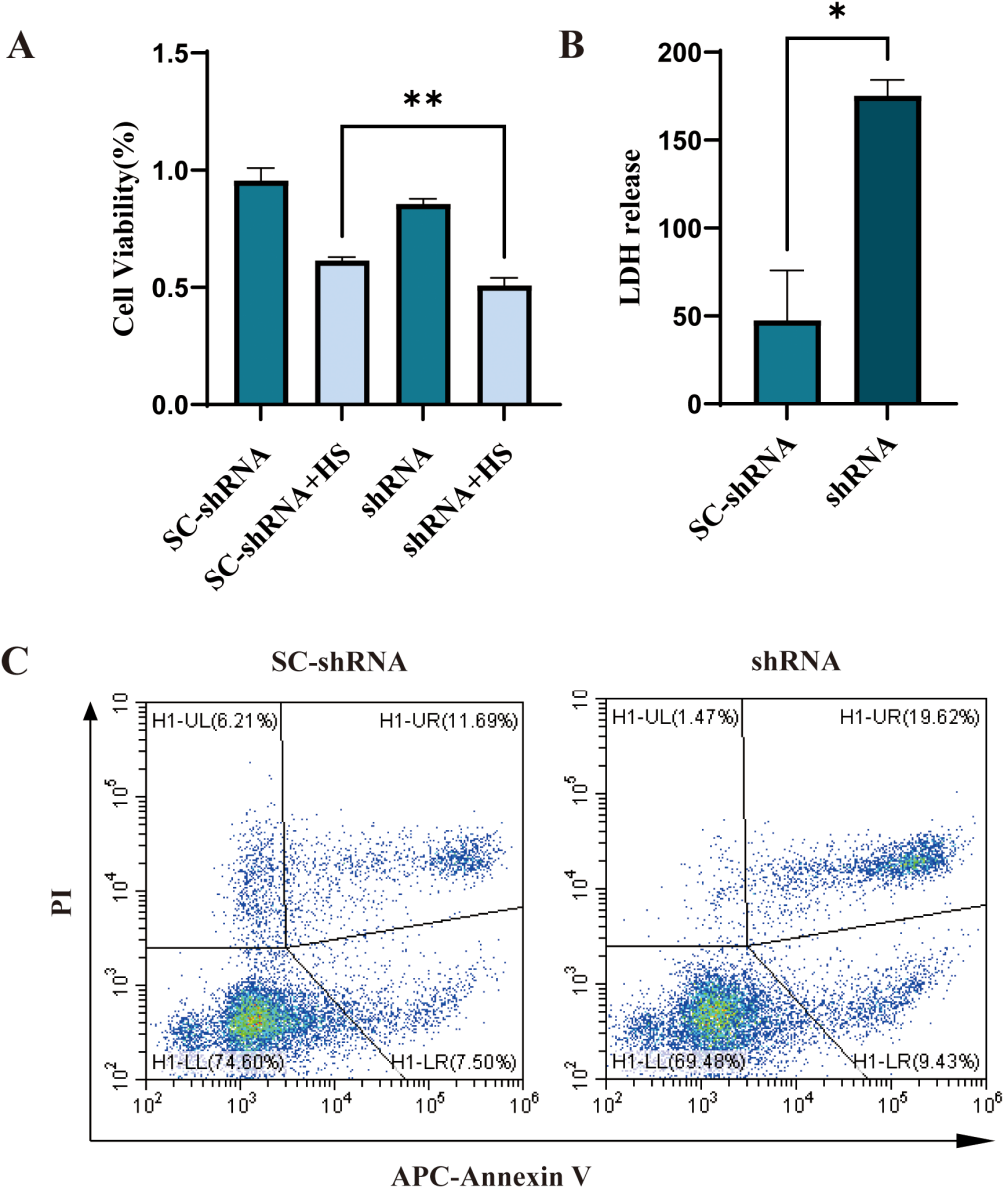
Knockdown of CD46 can induce apoptosis of OSCC cells: **(A)** The apoptosis of CAL-27 cells infected with SC-shRNA and shRNA and co-cultured with 20% human serum (HS) for 2h was compared (**P < 0.01). **(B)** Comparison of LDH release in supernatant (*P < 0.05). Apoptosis was detected by flow cytometry.

The complement binding assay results showed that the shRNA group’s cell survival rate was significantly lower than that of the SC-shRNA group, indicating that CD46 could inhibit complement activation, protect tumor cells from complement cleavage and promote tumor escape. Knockdown of CD46 can promote the apoptosis of OSCC (**Figure 4A**) (*P* < 0.01). The results of human lactate dehydrogenase (LDH) enzyme-linked immunosorbent assay showed that the LDH content in the supernatant of SC-shRNA infected cells was lower than that of shRNA infected cells, and the comparison between groups (**Figure 4B**) (*P* < 0.05). In order to further prove the relationship between CD46 and OSCC cell apoptosis, Annexin V-APC/PI cell apoptosis detection kit was used to detect cell apoptosis by flow cytometry. The results showed that after interfering with CD46, the percentage of early apoptotic cells increased significantly (**Figure 4C**), (*P* < 0.01). Consistent with the above results.

### 3.5 Analysis of western blot results and autophagosome detection

**Figure 5:**
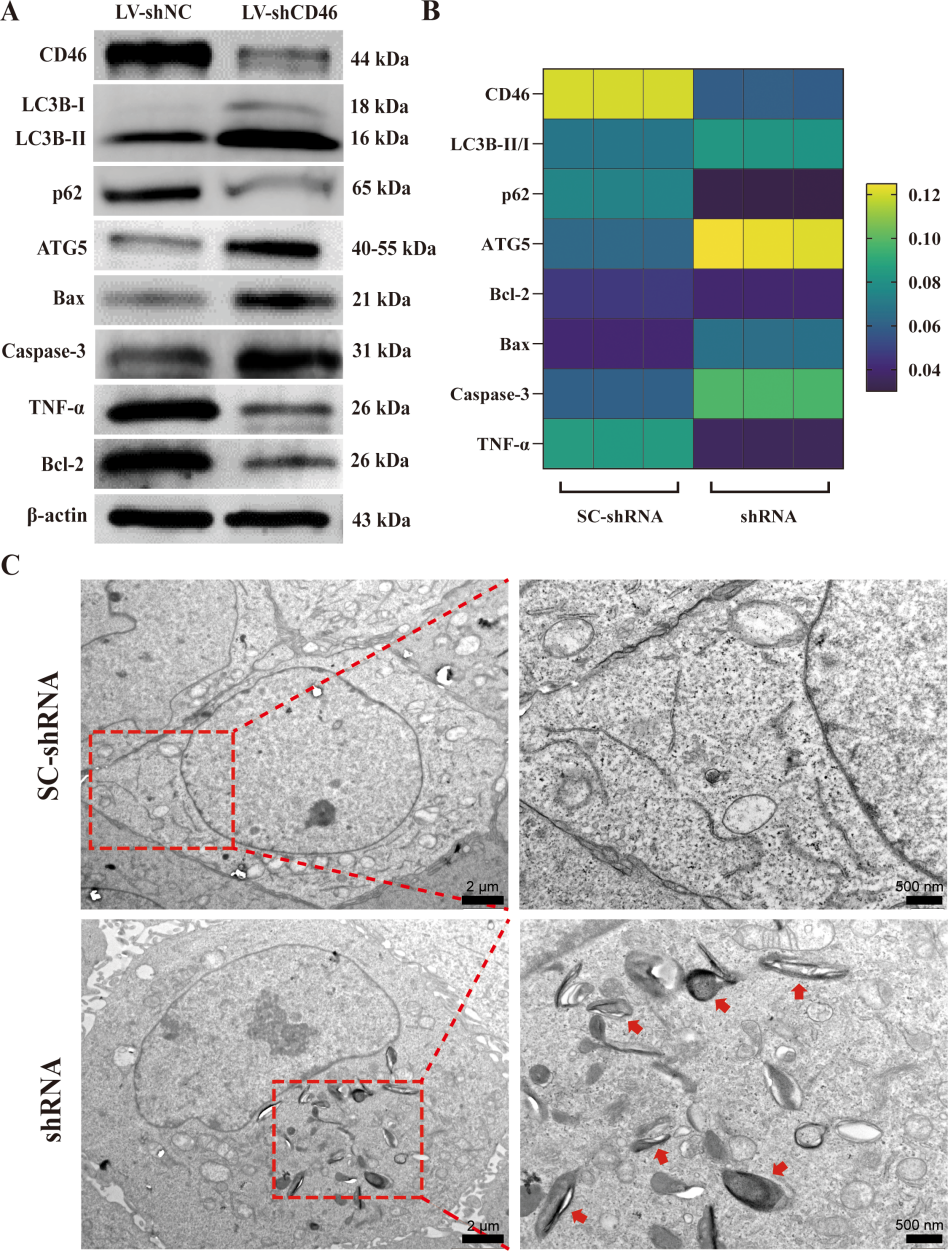
The relationship between CD46 and OSCC autophagy and apoptosis: **(A)** Western blot was used to analyze the expression of autophagy and apoptosis proteins in CAL-27 after SC-shRNA and shRNA treatment. **(B)** Heat map of various protein expression changes. **(C)** Representative TEM images of SC-shRNA and shRNA treatment respectively describe autophagosomes *in vitro*, with arrows indicating autophagosomes.

The results of Western blot showed that the expression of CD46 in the shRNA group was lower than that in the SC-shRNA group, the expression of autophagy proteins LC3B and ATG5 was increased. During autophagy, the microtubule-associated protein LC3B-I is converted to LC3B-II, a membrane-bound form that serves as a hallmark of autophagosome formation. The LC3B-II/I ratio is widely recognized as an indicator of autophagic activity. To distinguish whether the accumulation of LC3B-II resulted from enhanced autophagy induction rather than impaired autophagosome-lysosome fusion, we concomitantly measured the expression of p62 (a selective autophagy substrate degraded upon lysosomal fusion). Western blot analysis revealed that the shRNA group exhibited a significant decrease in the LC3B II/I ratio compared to the SCshRNA group, accompanied by a marked increase in p62 protein levels. In addition, the expression of apoptotic proteins Bax, Caspase-3 was increased, and the expression of anti-apoptotic protein Bcl-2 and tumor necrosis factor TNF-α was decreased, further indicating that inhibition of CD46 can promote OSCC apoptosis, which provides more powerful evidence for CD46 as a target for the treatment of OSCC (**Figure 5A, B**) (*P* < 0.05). Furthermore, we demonstrated that CD46 influenced autophagy *in vitro*. TEM revealed autophagosomes characterized by double-membrane boundaries surrounding mitochondria or other abundant cellular organelles. Notably, the number of autophagosomes was significantly increased in the shRNA group compared to the SCshRNA group (**Figure 5C**).

### 3.6 *In vivo* experiments: Effect of CD46 interference on autophagy and apoptosis of LC3B and ATG5 in tumor-bearing mice

**Figure 6:**
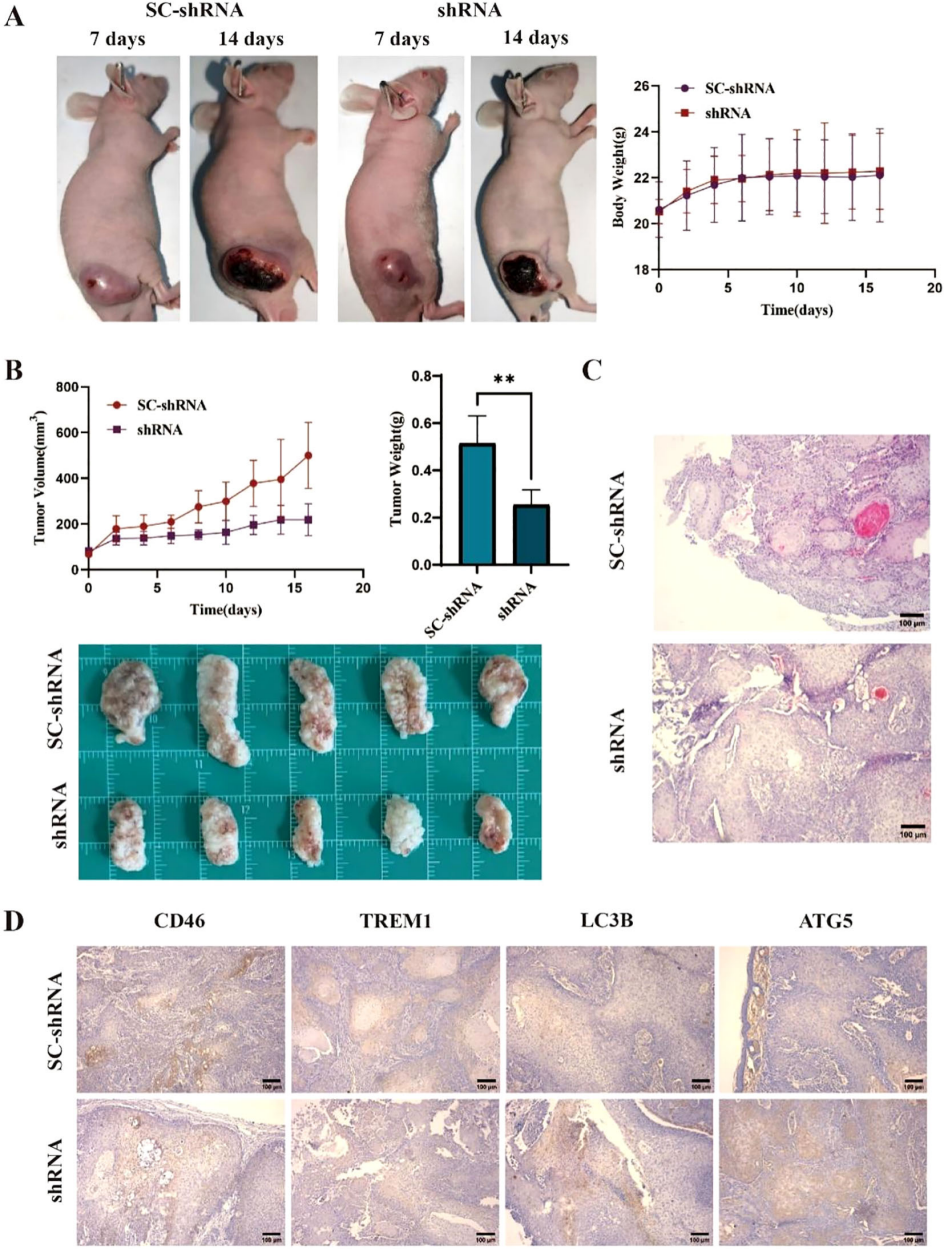
Knockdown of CD46 inhibits tumor growth in mice by inducing apoptosis: **(A)** Tumor comparison of CAL-27 treated with SC-shRNA and shRNA at 7 days and 14 days after successful tumor formation. **(B)** Tumor volume changes and weight statistics in different groups (**P < 0.01). **(C)** The results of HE staining in the two groups (10 ×). **(D)** Immunohistochemistry was used to detect the expression of CD46, TREM1, LC3B and ATG5 on the surface of tumors in different groups (10 ×).

On the 7th day after tumor cell injection, all mice were successfully implanted. The body weight and tumor diameter of mice were measured every other day. The results showed that the tumor size of the shRNA group was significantly smaller than that of the SC-shRNA group (**Figure 6A**) (*P* < 0.05). The mice were sacrificed at 2 weeks, and the tumors were weighed and photographed. The results showed that the tumor size of the shRNA group was smaller than that of the SC-shRNA group (**Figure 6B**) (*P* < 0.01). HE staining further confirmed the success of tumor implantation (**Figure 6C**). The results of immunohistochemistry showed that the expression of CD46 and TREM1 in the shRNA group was lower than that in the SC-shRNA group, and the expression of LC3B and ATG5 was higher than that in SC-shRNA group (**Figure 6D**) (*P* < 0.01). This indicates that CD46 can promote tumor proliferation and escape by inhibiting the activation of autophagy apoptotic genes.

## 4 Discussion

CD46 is a membrane-type complement regulatory protein (mCRP), expressed on various cell surfaces and is a key regulator of complement activation cascades. It can cleave C3b to produce C3bi and C3f and cleave C4b to produce C4d and C4c (13, 14). It is also a pathogen magnet effector molecule (15). On the one hand, it can protect tumor cells from complement cleavage and promote tumor escape by regulating the complement activation system (16, 17). On the other hand, it can form a tumor microenvironment by inhibiting tumor cell autophagy and promoting inflammatory response (18). TREM1 is a kind of myeloid cell surface receptor which is involved in regulating the expression level of macrophages. It is positively correlated with the secretion of inflammatory factors and participates in the formation of inflammatory tumor microenvironment (18–20). Studies have shown that CD46 and TREM1 are highly expressed on the surface of a variety of tumor cells throughout the body. Various experimental results support that their increased expression is associated with malignant transformation and metastasis of tumors (13, 21, 22).

Autophagy is a mechanism of cell self-protection classified as “programmed cell death.” The correlation between autophagy and tumor apoptosis has become a hot topic in medicine, and its detailed mechanism needs to be further explored (23–27). Based on this, this study aims to explore the regulatory effects of CD46 and TREM1 on OSCC autophagy and apoptosis genes and provide new ideas for the molecular mechanism and targeted therapy of OSCC. There is no detailed report on home or abroad. In this study, immunohistochemistry was used to observe the expression of CD46 and TREM1 genes on the surface of tissues. The results showed that CD46 and TREM1 genes were overexpressed on the surface of cancer tissues. The expression of CD46 was more significant, while the expression levels of autophagy apoptosis genes LC3B and ATG5 were decreased. This indicates that CD46 and TREM1 promote tumor proliferation and metastasis by inhibiting autophagy and apoptosis during OSCC development. This study focuses on CD46, the CD46 gene in OSCC cells was interfered by CD46 shRNA, and the expression of LC3 B and ATG5 genes before and after CD46 interference was further compared by qRT-PCR and Western blot. The results showed that the expression of autophagy apoptosis genes LC3B and ATG5 increased significantly with the decrease of CD46. The expression of apoptosis-related molecules Bax and Caspase-3 was significantly increased, while the expression of anti-apoptotic Bcl-2 was decreased. The results of flow cytometry and LDH assay were highly consistent. TREM1 shRNA interference will be studied in subsequent experiments.

*In vivo* experiments of tumor-bearing animal models showed that the tumor growth rate and volume of the CD46 interference group were significantly lower than those of the control group (*P* < 0.01), indicating that the expression of LC3B and ATG5 on the surface of the interference CD46 tumor was increased. The apoptosis signal of OSCC cells was enhanced.

## 5 Conclusion

CD46 and TREM1 are highly expressed on the surface of OSCC cells, which can protect tumor cells from complement dissolution and promote the proliferation of OSCC cells by inhibiting complement activation; otherwise, CD46 and TREM1 inhibit the apoptosis of OSCC cells by inhibiting the expression of LC3B and ATG5 autophagy and apoptosis genes, which is beneficial to the escape of oral squamous cell carcinoma cells; therefore, CD46 can be used as a new target for the diagnosis and treatment of OSCC. This study provides new ideas for the molecular mechanism and targeted therapy of OSCC. It has important theoretical significance and clinical application value.

## Data Availability

The original contributions presented in the study are included in the article/**Supplementary Material**. Further inquiries can be directed to the corresponding author/s.
