# Supplementary material for: CD46 TREM1 regulates the autophagy marker LC3B ATG5 in oral squamous cell carcinoma
Source: Front Oncol. 2025 May 22;15:1579282. doi: 10.3389/fonc.2025.1579282 (PMC12137063; doi:10.3389/fonc.2025.1579282)
Supplement: Supplementary file 1 [file DataSheet1.docx]

**Supporting Information**

**CD46 TREM1 regulates the autophagy marker LC3B ATG5 in oral squamous cell carcinoma**

Xiaodan Liu^1,†^, Fumin Zheng^2,3,†^, Meihua Gao^2^, Yuanfei Wang^2^, Xiaofang Lv^2,3^, Beibei Cong^2^*, Wanchun Wang^2^*

*****Correspondence:

[xinruo1986@163.com](mailto:xinruo1986@163.com) (Beibei Cong)

[kqwwch@126.com](mailto:kqwwch@126.com) (Wanchun Wang)

†These authors contributed equally to this work.

| Table S1 The clinical information and sample size for TCGA CRC dataset | | | | |
| --- | --- | --- | --- | --- |
| **Characteristics** | **Living (n=305)** | **Deceased (n=225)** | **Total (n=530)** | ***P*** |
| **Cancer type** |  |  |  |  |
| Head and Neck region | 305(57.55%) | 225(42.45%) | 530(100.00%) |  |
| **Age** |  |  |  |  |
| Mean±SD | 59.46±11.08 | 62.88±12.71 | 60.91±11.91 |  |
| Median[min-max] | 60.00[19.00,85.00] | 63.00[24.00,90.00] | 61.00[19.00,90.00] |  |
| **Gender** |  |  |  | 0.02 |
| Female | 70(13.21%) | 73(13.77%) | 143(26.98%) |  |
| Male | 235(44.34%) | 152(28.68%) | 387(73.02%) |  |
| **Histological_type** |  |  |  | 0.18 |
| Head & Neck Squamous Cell Carcinoma | 297(56.04%) | 222(41.89%) | 519(97.92%) |  |
| Head & Neck Squamous Cell Carcinoma Basaloid Type | 8(1.51%) | 2(0.38%) | 10(1.89%) |  |
| Head & Neck Squamous Cell Carcinoma, Spindle Cell Variant | 0(0.0e+0%) | 1(0.19%) | 1(0.19%) |  |
| **Stage** |  |  |  | 9.10E-04 |
| Stage I | 23(5.02%) | 4(0.87%) | 27(5.90%) |  |
| Stage II | 48(10.48%) | 29(6.33%) | 77(16.81%) |  |
| Stage III | 53(11.57%) | 29(6.33%) | 82(17.90%) |  |
| Stage IV | 137(29.91%) | 135(29.48%) | 272(59.39%) |  |
| **Weight** |  |  |  |  |
| Mean±SD | 184.29±161.22 | 166.45±122.73 | 177.93±148.53 |  |
| Median[min-max] | 140.00[10.00,870.00] | 145.00[10.00,600.00] | 140.00[10.00,870.00] |  |
| Values are n(%), unless otherwise noted. | | | | |

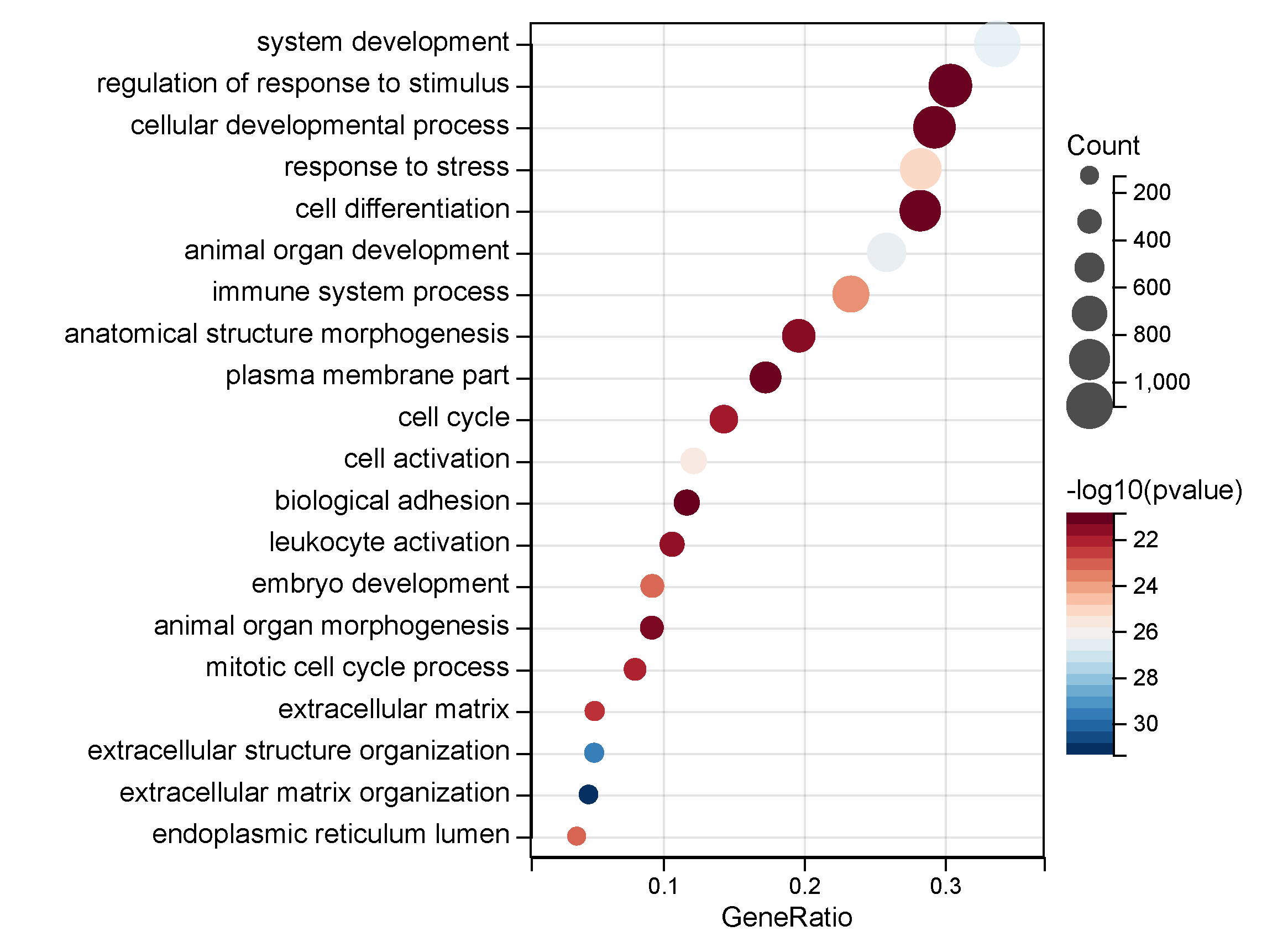


Fig S1: Functional enrichment analysis of upregulated genes via Gene Ontology (GO). Dot size corresponds to the count of genes enriched in each ontology term, while color gradients indicate the statistical significance of enrichment (darker shades represent lower P-values).


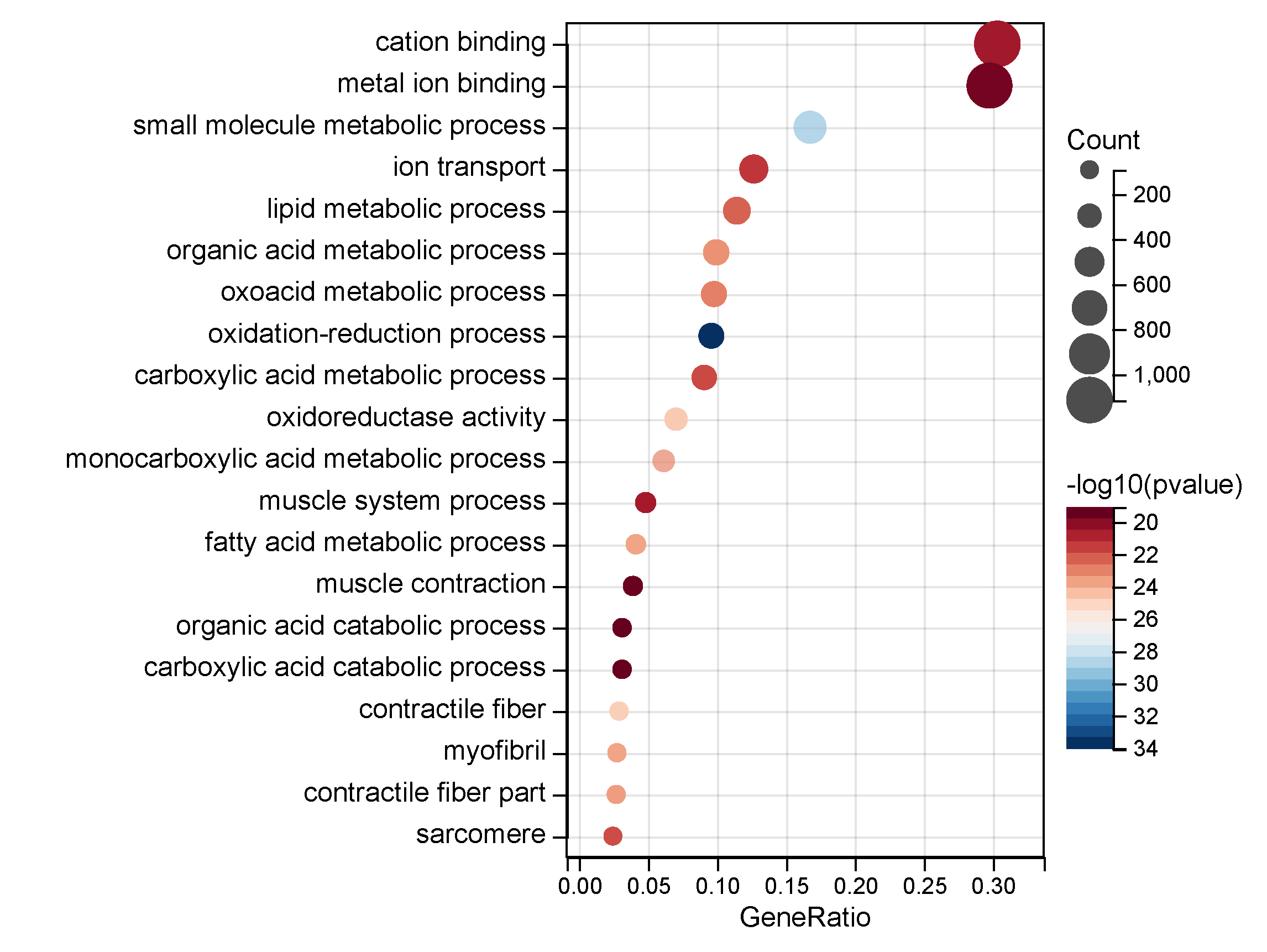


Fig S2: Functional enrichment analysis of downregulated genes via Gene Ontology (GO). Dot size corresponds to the count of genes enriched in each ontology term, while color gradients indicate the statistical significance of enrichment (darker shades represent lower P-values).
